# Supplementary material for: Development and evaluation of a loop-mediated isothermal amplification (LAMP) assay for the detection of Tomato brown rugose fruit virus (ToBRFV)
Source: PLoS One. 2020 Jun 24;15(6):e0230403. doi: 10.1371/journal.pone.0230403 (PMC7313975; doi:10.1371/journal.pone.0230403)
Supplement: S1 Raw images — (PDF) [file pone.0230403.s002.pdf]

S1_raw_images: Fig. 2D

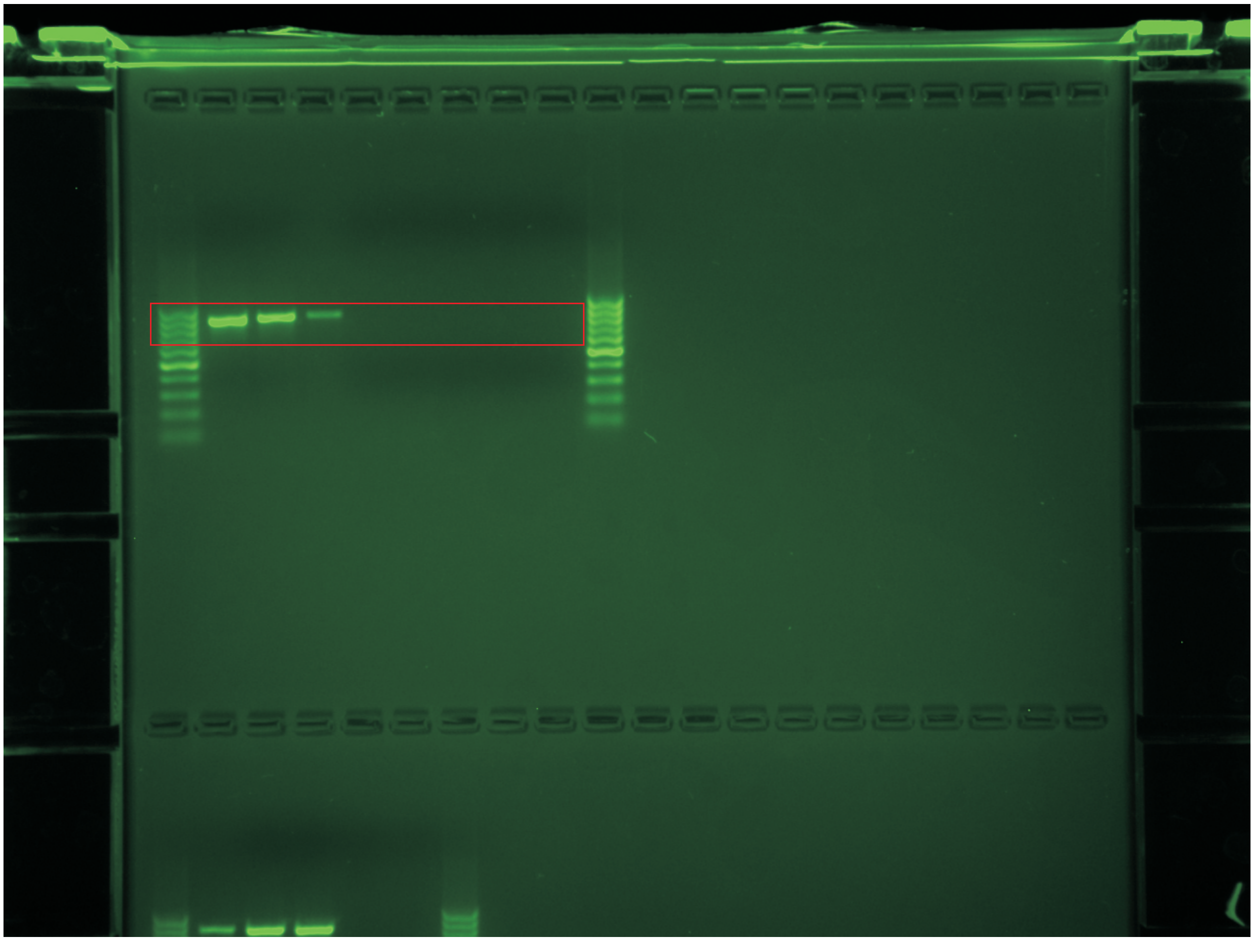

The marker is Omega 100bp DNA Ladder (https://www.omegabiotek.com/product/100bp-dna-ladder)
The photo was taken with Bio-Rad Gel Doc XR

S1_raw_images: Fig. 4B

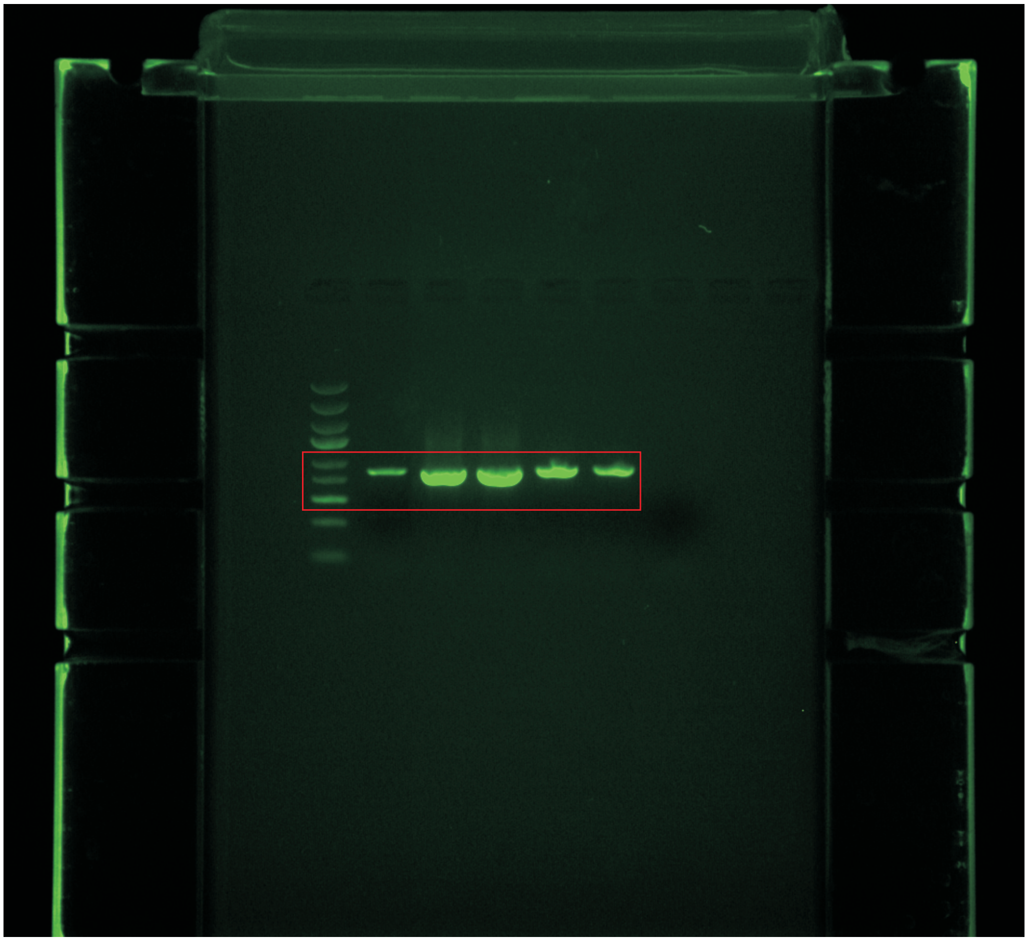

The marker is GeneRuler express DNA ladder
(https://www.thermofisher.com/order/catalog/product/SM1553#/SM1553)
The photo was taken with Bio-Rad Gel Doc XR

S1_raw_images: Fig. 4D

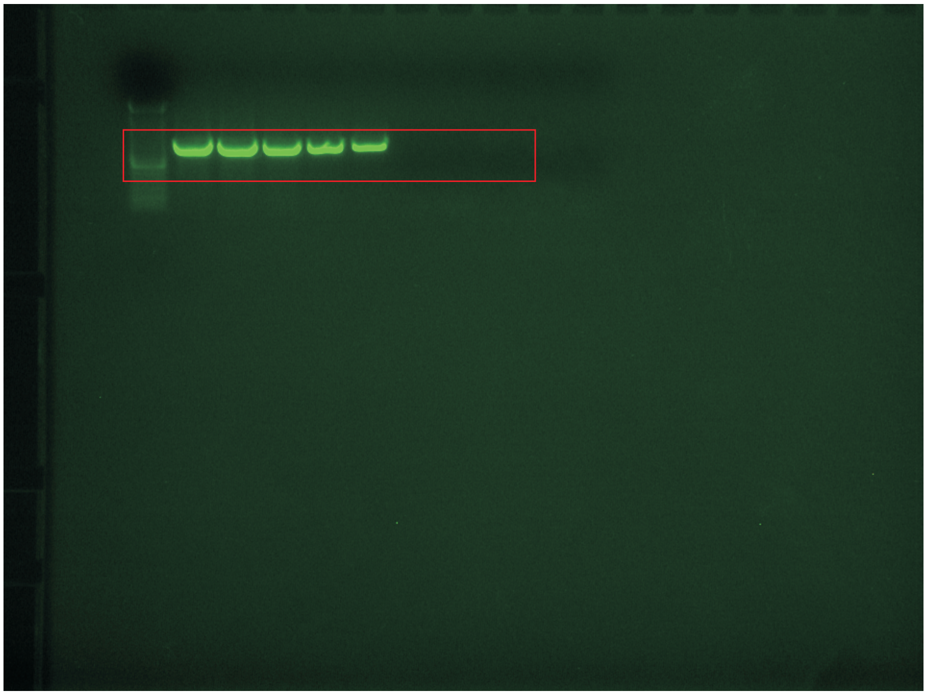

The marker is TrackIt 100 bp DNA ladder
(https://www.thermofisher.com/order/catalog/product/10488058#/10488058)
The photo was taken with Bio-Rad Gel Doc XR

S1_raw_images: Fig. 5B

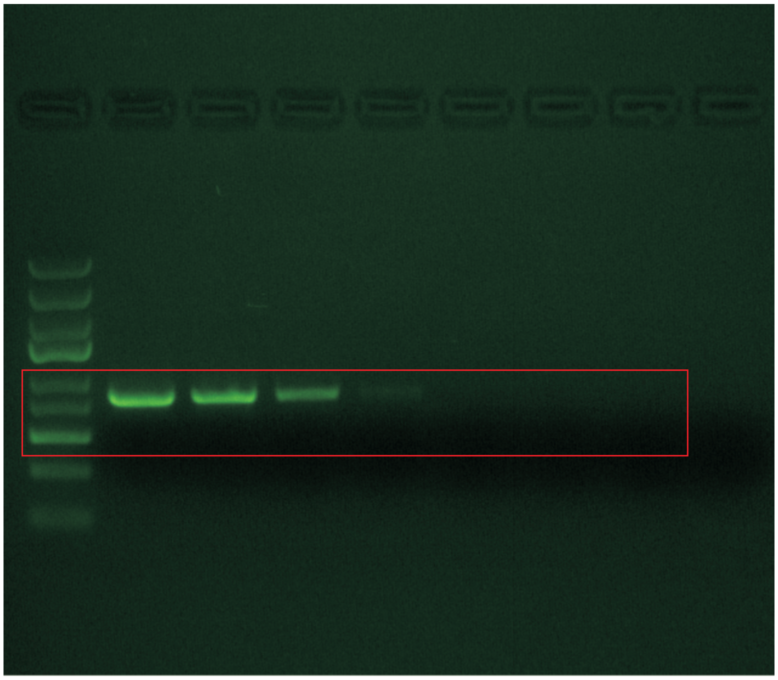

The marker is GeneRuler express DNA ladder
(https://www.thermofisher.com/order/catalog/product/SM1553#/SM1553)
The photo was taken with Bio-Rad Gel Doc XR
